# Thymic rejuvenation via *FOXN1*-reprogrammed embryonic fibroblasts (FREFs) to counteract age-related inflammation

**DOI:** 10.1172/jci.insight.140313

**Published:** 2020-09-17

**Authors:** Jiyoung Oh, Weikan Wang, Rachel Thomas, Dong-Ming Su

**Affiliations:** Department of Microbiology, Immunology, & Genetics, University of North Texas Health Science Center, Fort Worth, Texas, USA.

**Keywords:** Aging, Immunology, Immunotherapy, T cell development

## Abstract

Age-associated systemic, chronic inflammation is partially attributed to increased self-autoreactivity, resulting from disruption of central tolerance in the aged, involuted thymus. This involution causally results from gradually decreased expression of the transcription factor *FOXN1* in thymic epithelial cells (TECs), whereas exogenous *FOXN1* in TECs can partially rescue age-related thymic involution. TECs induced from *FOXN1*-overexpressing embryonic fibroblasts can generate an ectopic de novo thymus under the kidney capsule, and intrathymic injection of naturally young TECs can lead to middle-aged thymus regrowth. Therefore, as a thymic rejuvenation strategy, we extended these 2 findings by combining them with 2 types of promoter-driven (*Rosa26*CreER^T^ and *FoxN1*Cre) Cre-mediated *FOXN1*-reprogrammed embryonic fibroblasts (FREFs). We engrafted these FREFs directly into the aged murine thymus. We found substantial regrowth of the native aged thymus with rejuvenated architecture and function in both males and females, exhibiting increased thymopoiesis and reinforced thymocyte negative selection, along with reduced senescent T cells and autoreactive T cell–mediated inflammation in old mice. Therefore, this approach has preclinical significance and presents a strategy to potentially rescue decreased thymopoiesis and perturbed negative selection to substantially, albeit partially, restore defective central tolerance and reduce subclinical autoimmune symptoms in elderly people.

## Abstract 2

Engrafting FOXN1-reprogrammed embryonic fibroblast directly into the aged murine thymus promoted regrowth of the native thymus with rejuvenated architecture and function.

## Graphical abstract

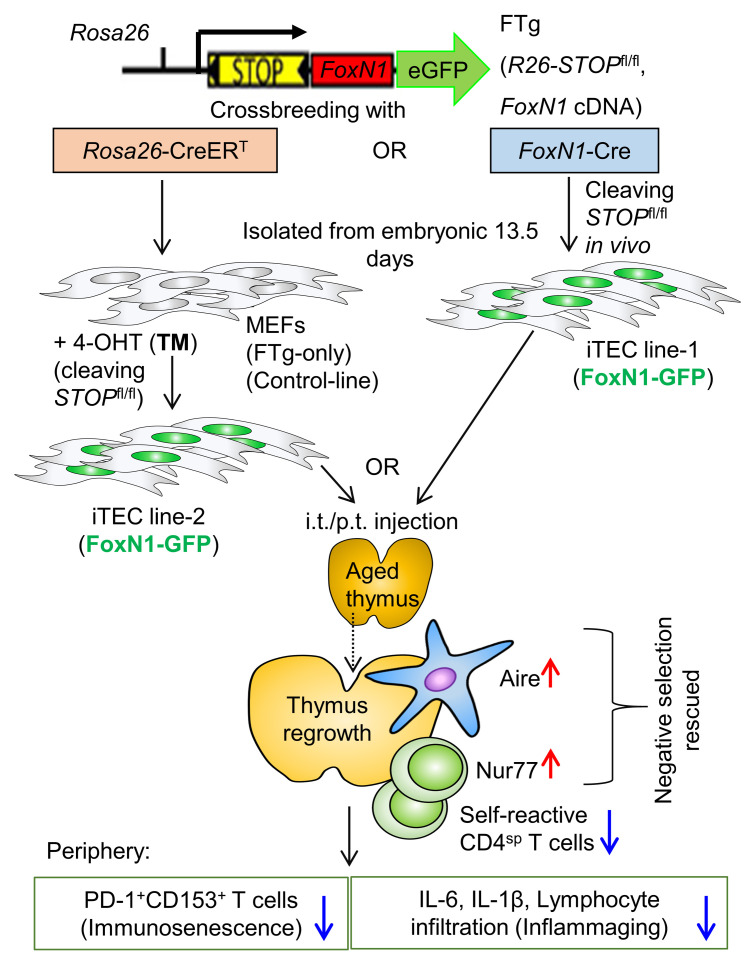

## Introduction

Age-related immune dysfunction is generally characterized by 2 extremes: immunosenescence (immune insufficiency) (1) and inflammaging (a chronic, persistent, sterile, systemic inflammation, partially owing to strong self-reactivity) (2, 3). These are antagonistic phenotypes; however, they actually comprise 2 sides of the same coin (4) and are associated with functional defects in the aged, atrophied thymus (5–8). Immunosenescence, unlike cultured cellular senescence, occurs at systemic levels exhibiting diminished immune reaction in response to antigen stimulations, mainly owing to contracted T cell receptor (TCR) repertoire diversity (9). This is primarily attributed to a decreased output of naive T cells from the aged, atrophied thymus (10) and expansion of monoclonal memory T cells in the periphery (detailed in our review) (11). Although inflammaging was originally attributed to somatic cell senescence-associated secretory phenotype (SASP) (12) and chronic innate immune activation (4, 13), the contribution of aged adaptive immune components, specifically self-reactive T lymphocytes, as a probable primary contributor has been recently determined (7, 13). The increased self-reactive T cells in elderly people are derived from perturbed central T cell tolerance establishment (6, 7, 14), owing to defects in negative selection and altered Treg generation (7, 15) in the aged, atrophied thymus.

During aging the thymus undergoes a progressive, age-related atrophy, or involution, and a key trigger is the primary defect in thymic epithelial cells (TECs), which is mainly attributed to gradually diminished expression of transcription factor forkhead box N1 (*FOXN1*) in TECs (16–18). Therefore, to ameliorate immunosenescence and reduce inflammaging through restoration of the aged T cell immune system, many studies have focused on targeting TECs in the aged thymus. Because reduction of the TEC-autonomous factor *FOXN1* is heavily implicated in onset and progression of age-related thymic involution, for rejuvenation of the aged thymus additional strategies concentrate on the *FOXN1*-TEC axis, although others exist, such as modulation of cytokines and growth and/or sex hormones (detailed in our review) (11). *FOXN1*-TEC axis strategies include *FoxN1*^EGFP/+^-knockin epithelial cells (19), newborn TEC-based intrathymic (i.t.) injection (20), inducible *FoxN1*-expressed mouse embryonic fibroblast–based (MEF-based) ectopic thymus generation (21), and genetically based rejuvenation via enhancement by exogenous *FoxN1* expression with *FoxN1* cDNA plasmid (16) and *FoxN1* Tg in TECs (22, 23). In addition, cytokine/growth factor-to-TEC-based rejuvenation strategies have been studied, including addition of mesenchymal cell-derived keratinocyte growth factor (KGF) (24), macrophage- and T lymphocyte-derived IGF-1 (25), thymic stromal cell-derived bone morphogenetic protein-4 (BMP4) (26, 27), and lymphoid tissue inducer cell-derived IL-22 (28). These factors are produced from cells of mesenchymal or hematopoietic origin, but target nonhematopoietic TECs associated with upregulating *FoxN1* expression in TECs. Finally, epigenetically based rejuvenation, via extracellular vesicles and exosomes extracted from young, healthy serum, has been shown to rejuvenate not only the peripheral T cell system, but also the thymus by enhancing *FoxN1* expression (29). Therefore, potential exists for rejuvenating thymic aging by primarily targeting the restoration of TEC homeostasis through rescuing age-related decreased *FoxN1* expression.

Among the available *FOXN1*-TEC axis therapies for thymic rejuvenation, 2 strategies are particularly attractive. One strategy is to aggregate induced *Rosa26(R26)*CreER^T^-mediated *FOXN1*-overexpressed MEFs (converting these cells into pseudo-TECs, termed “induced TECs” or “FREFs”) along with early-stage thymocytes and fetal mesenchymal cells to build an ectopic thymus under the kidney capsule of adult mice (21). Because these cells are not actual TECs, but embryonic fibroblasts with FoxN1 overexpression, we term them “FoxN1-reprogrammed embryonic fibroblasts” (FREFs). This de novo ectopic thymus produced functional T cells. However, one limitation is that the aged, native thymus remains in the host, releasing self-reactive T cells that still contribute to inflammaging. The other strategy is an intrathymic injection of freshly isolated newborn TECs (nonmanipulated TECs), in which *FoxN1* is normally highly expressed into the native thymus of middle-aged mice (20). This leads to restoration of thymopoiesis. However, collection of fresh newborn TECs is not feasible when considering translating this rejuvenation strategy to humans, and isolation of fresh TECs without thymocyte contamination is very difficult considering that TECs and their progenitors comprise a miniscule portion of the thymus (30). Therefore, these promising thymic rejuvenation strategies for development of a practical therapy contain several limitations.

Fortunately, fibroblasts, which could be very easily isolated from human patients, can be engineered and/or reprogrammed to overexpress *FOXN1* for induced TECs/FREFs (iTECs/FREFs) for intrathymic injection. Based on these scientific premises, we expanded on these 2 findings and applied them to develop a novel thymic rejuvenation strategy. We directly engrafted FREFs into the aged, native thymus to rejuvenate thymic function and assessed this in a mouse model, by using MEFs from our engineered *STOP*^fl/fl^-*FoxN1-*Tg (FTg) mouse allele (31, 32) (Supplemental Figure 1; supplemental material available online with this article; https://doi.org/10.1172/jci.insight.140313DS1), mediated by 2 types of promoter-driven (*R26-*CreER^T^ and *FoxN1*Cre) Cre-recombinase.

We found that the engrafted FREFs drove regrowth of the aged thymuses in both male and female mice with increased thymopoiesis and improved thymic architecture. These results led to a reinforcement of thymocyte negative selection in the native, aged thymus, thereby attenuating autoreactive T cell–mediated inflammaging phenotypes and reducing senescent T cells in old mice. Although the native, aged thymus cannot fully return to young levels in our system, this is a promising thymic rejuvenation strategy with preclinical significance to counteract inflammaging.

## Results

### Preparation and characterization of FREFs.

**Figure 1:**
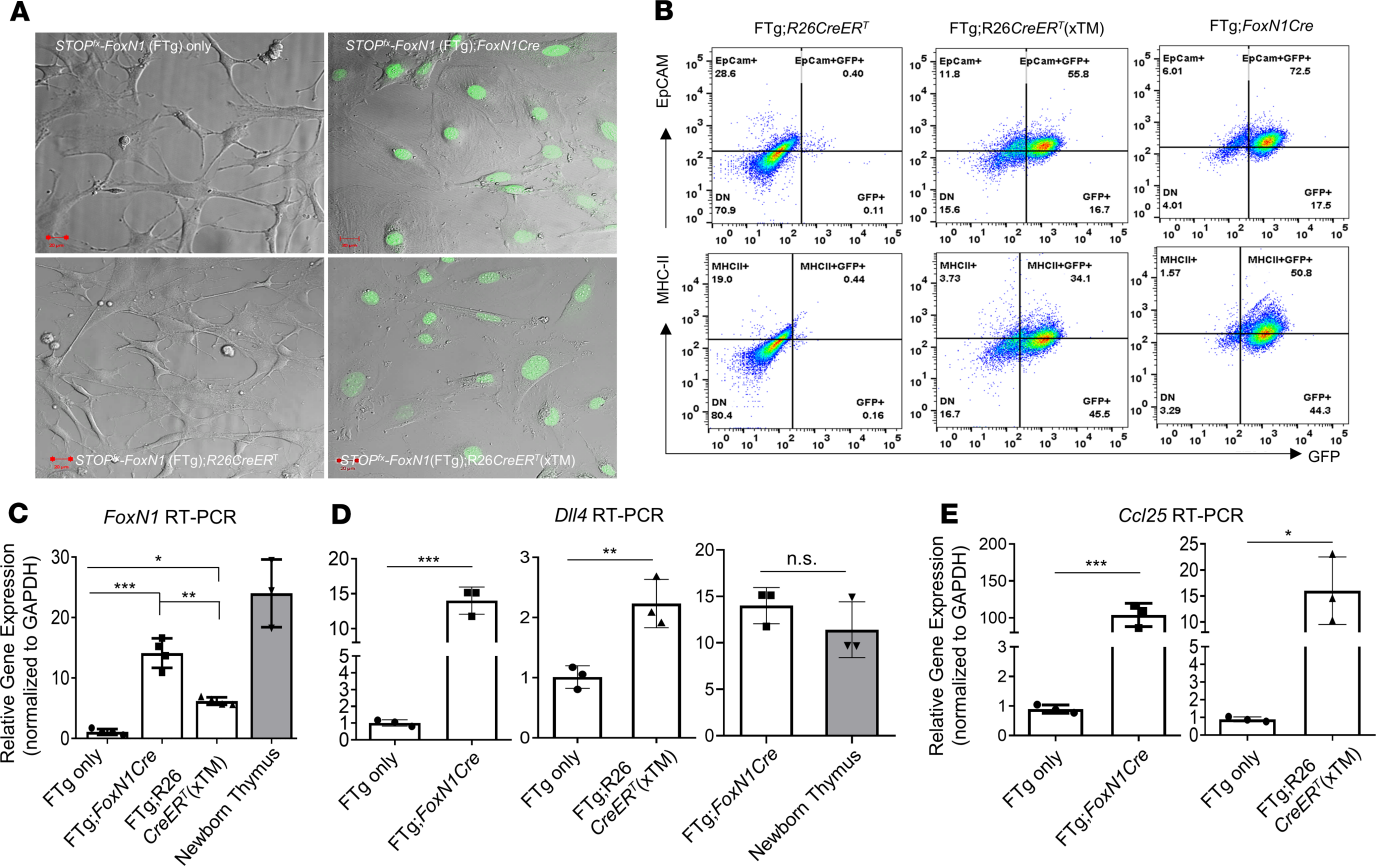
Preparation and characterization of MEFs and FREFs. Mouse embryonic fibroblasts (MEFs) were isolated via trypsinized digestion from E13.5 embryonic mice and cultured in plates with or without 4-hydroxy tamoxifen (xTM). (**A**) Representative live images from confocal microscopy show MEFs expressing GFP, which represents exogenous FoxN1 (right) and was driven by either endogenous *FOXN1*-carried Cre-recombinase at 3′UTR (FTg:*FoxN1Cre*; top) or *R26*-carried CreER^T^ treated with TM (FTg:*R26CreER*^T^ xTM; bottom) and panels without GFP (left) owing to either no Cre transgene or no active Cre. Scale bar: 20 μm. (**B**) Representative flow cytometric dot plots (EpCAM vs. GFP: top; and MHC-II vs. GFP: bottom), in which MEFs expressing GFP (FTg:*R26CreER*^T^ xTM and FTg:*FoxN1Cre*) are termed “FREFs” (in red, middle and right panels), compared with MEFs that did not express GFP (FTg:*R26CreER*^T^ owing to Cre inactivated without TM treatment, left panels). (**C**) Summarized gene (*FoxN1*) expression (via reverse transcription PCR; RT-PCR) in cells of 4 groups: (a) FTg-only (without Cre), (b) FTg:*FoxN1Cre* (Cre expression was endogenously turned on in *FoxN1^+^* cells), (c) FTg:*R26CreER*^T^ xTM (Cre was activated via TM induction), and (d) a newborn thymus control group. One-way ANOVA Newman-Keuls multiple-comparisons test was used. (**D**) Summarized gene (*Dll4*) and (**E**) (*Ccl25*) expression (via RT-PCR) in cells of 4 groups as in **C** (these are 2-and-2 group comparisons). Student’s *t* test was used to determine statistical significance. *P* values are shown between every 2 groups, and all *P* values represent the mean ± SD. Each symbol represents cells from an individual embryonic sample. Statistical significance levels were set at *P < 0.05, **P < 0.01, and ***P < 0.001. n.s., not significant.

A previous report demonstrated that enforced *FOXN1* expression in MEFs from embryos generated by crossbreeding of *STOP*^fl/fl^-FTg and *R26-CreER*^T^ mice induced epithelial characteristics in fibroblasts (21). Because we generated similar *STOP*^fl/fl^-FTg (exogenous *FoxN1* cDNA driven by *R26* promoter and carrying a GFP reporter, termed “FTg”) mice (DNA construct is shown in Supplemental Figure 1) (31, 32), we crossbred these mice with either *R26-CreER*^T^ or *FoxN1-Cre* mice to generate FTg:*R26CreER*^T^ and FTg:*FoxN1Cre* embryonic mice, respectively. To investigate *FoxN1-Cre–*mediated activation of FTg, we first analyzed GFP-positive cell expression in freshly isolated FTg:*FoxN1Cre* MEF cells (isolated from embryonic day 13.5, termed “E13.5, mice”). We found that exogenous *FoxN1* is turned on when endogenous *FoxN1* is activated via Cre-driven deletion of the STOP codon, and these cells can continuously express GFP for 48 hours during in vivo cell culture (Supplemental Figure 2A). We confirmed MEF epithelial characteristics from 2 different promoter-driven *FoxN1*-expressing lines (Figure 1). Using nuclear localization signal-enhanced GFP as an indicator of exogenous *FoxN1* expression (31, 32) in the cultured MEFs, we found MEFs from FTg only (without any Cre-Tg) and FTg:*R26CreER*^T^ without addition of tamoxifen (xTM, 4-hydroxy TM [4-OHT]) did not express GFP (Figure 1A) attributed to lack of activated Cre, whereas FTg:*FoxN1Cre* (TM not required) and FTg:*R26CreER*^T^ lines treated with TM for 48 hours showed GFP expression (Figure 1, A and B). We also found that MEFs with greatly increased exogenous *FoxN1* expression from FTg:*FoxN1Cre* and xTM mice showed TEC identifying markers (EpCAM^+^ and MHC-II^+^ cells in the GFP^+^ population) (Figure 1B), but not MEFs from FTg:*R26CreER*^T^ without addition of TM (Figure 1B).

Exogenous *FoxN1* mRNAs were increased in the 2 Cre-activated groups (Figure 1C, 2 middle bars. In addition, some TEC functional molecules, which are key effectors in promoting thymocyte development, such as Notch ligand *Dll4* and thymus-expressed chemokine ligand *Ccl25*, were increased in MEFs with activated *Cre*-Tg (Figure 1, D and E). Notably, both exogenous *FoxN1* and effector molecule expression increased in FREFs; however, their increased levels in these pseudo-TECs were still lower or similar to their expression in the natural newborn thymus, when these molecules should be normally highly expressed (Figure 1, C and D, gray bars). In addition, *FoxN1*Cre-mediated expression of exogenous *FoxN1* and effector molecules in the FTg:*FoxN1Cre* line was higher than in the *R26*CreER^T^-mediated line (Figure 1C). This is probably attributed to *Cre-*Tg turning on via endogenous *FoxN1* in vivo, which is activated at embryonic day 11.25 in the thymus (33) and potentially in the embryonic day 12.5 (E12.5) skin (34) or alternatively at low levels in the E13.5 skin (21) (Supplemental Figure 2A) during the organogenesis of B6 mice. This in vivo endogenous *FoxN1*-induced exogenous *FoxN1* expression is 48 hours earlier than in vitro TM-induced expression in the FTg:*R26CreER*^T^ line. Together, Cre-induced expression of exogenous/reprogrammed *FoxN1* and TEC functional molecules in MEFs conferred TEC characteristics to these MEFs. Therefore, these MEFs were termed “FREFs.”

### Intrathymic/perithymic transplantation of FREFs drove aged thymus regrowth.

**Figure 2:**
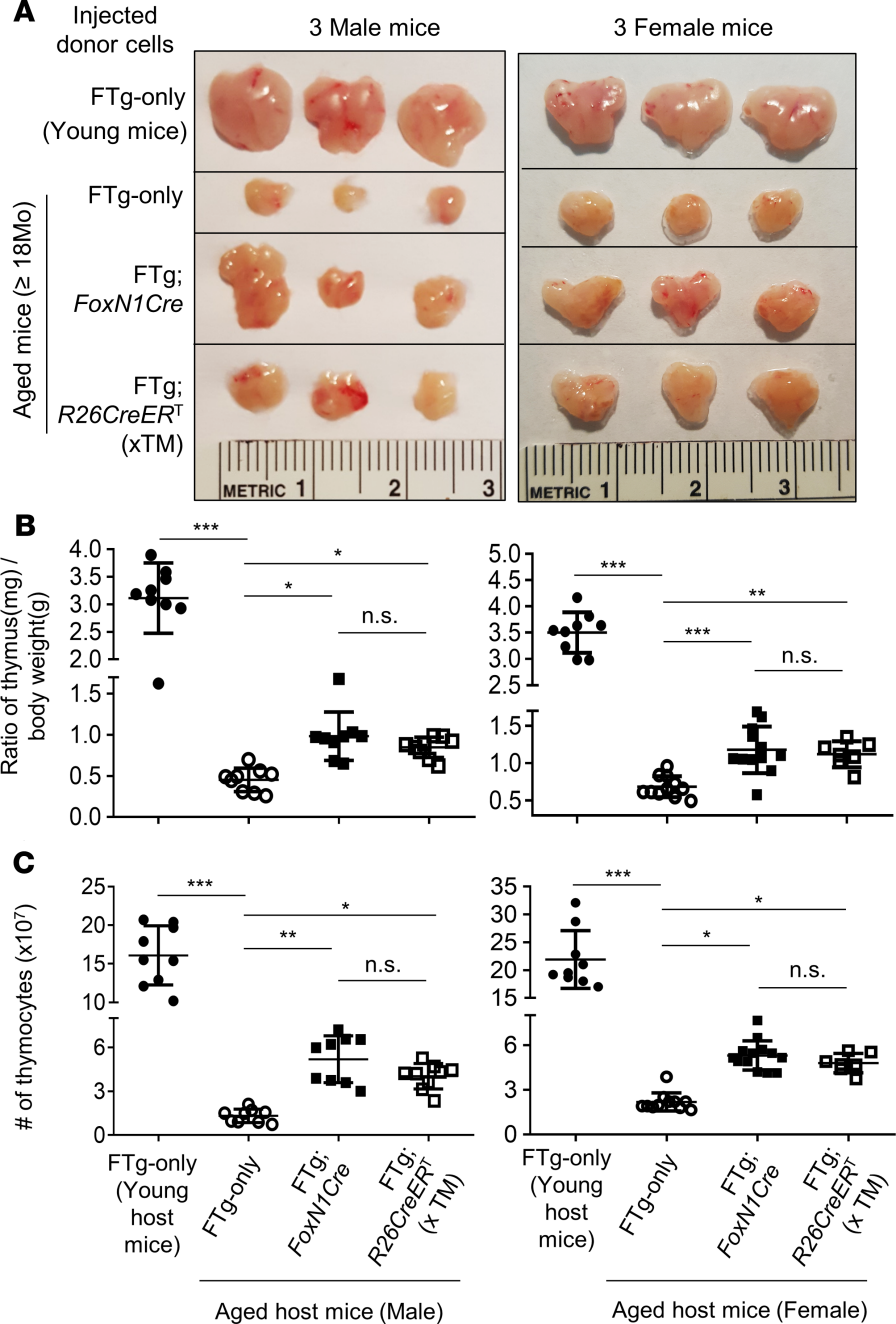
Transplantation of FREFs drove regrowth of the aged thymus in both male and female mice. Naturally aged mice (WT, ≥18 months old at cellular transplantation; 20–21 months old at analysis) were intra-/perithymically (i.t./p.t.) transplanted with FTg-only MEFs or either of 2 promoter-driven exogenous *FoxN1*-expressing FREFs; 1 group of young mice served as a control. Forty-five days after engraftment, the thymic mass was analyzed. (**A**) Representative images of the thymuses engrafted with donor cells. (**B**) Ratios of thymus/body weight. (**C**) Results of absolute thymocyte numbers per thymus from donor cell–engrafted aged male and female mice (1 young group, left, served as control). All of the data are from 3 independent experiments. Each symbol represents mice number per group. All *P* values represent the mean ± SD and compared with 1-way ANOVA Newman-Keuls multiple-comparisons tests. *P < 0.05, **P < 0.01, ***P < 0.001. n.s., not significant.

Given the data shown by another group that i.t. injection of fetal thymic cells, containing young TECs with high levels of *FoxN1* expression, into middle-aged (9–12 months old) mice drove recipient thymus growth and increased T cell production (20), we determined whether our FREFs could yield similar outcomes in fully aged (>18 months old) mice. We first examined thymus regrowth and thymopoiesis of aged mice (≥18 months old at the time of injection and 19–20 months old at the time of analysis) after i.t./perithymic (p.t.) transplantation of FREFs. Our results show that transplantation of FREFs drove aged mouse thymus regrowth (Figure 2) exhibited by increased thymic size, weight, and thymocyte numbers, compared with age-matched, control cell–treated (FTg-only) mouse thymus (Figure 2, A–C). These changes were the same in both male and female mice. Although these improvements did not reach the same levels as in the young mice (Figure 2, A–C), it improved compared with the naturally aged group without transplantation of FREFs (transplantation of FTg-only MEFs served as a negative control allowing for the same surgical stress as the FREF-engrafted groups).

Overall, our FREFs better resemble newborn TECs and efficiently drive the atrophied thymus regrowth and rejuvenation of thymopoiesis in aged (≥18 months old) mice. It appears that the efficacy from both FREF lines were generally similar; however, endogenous *FoxN1* promoter-driven Cre was slightly better than *R26* promoter-driven CreER^T^ (xTM) (Figure 2C). This could be explained by the fact that although *R26*CreER^T^ is turned on in vitro during the culture with TM induction, this occurs 48 hours later than the in vivo activation of *FoxN1*Cre. Expression of the effector molecules in the FTg:*R26*CreER^T^ line was lower than the FTg:*FoxN1*Cre line (Figure 1, C–E); however, they could become equivalent after injection into the host thymuses, considering that the effector molecules probably increase only to a homeostatic plateau.

### Grafted FREFs rejuvenated thymic architecture in aged mice.

**Figure 3:**
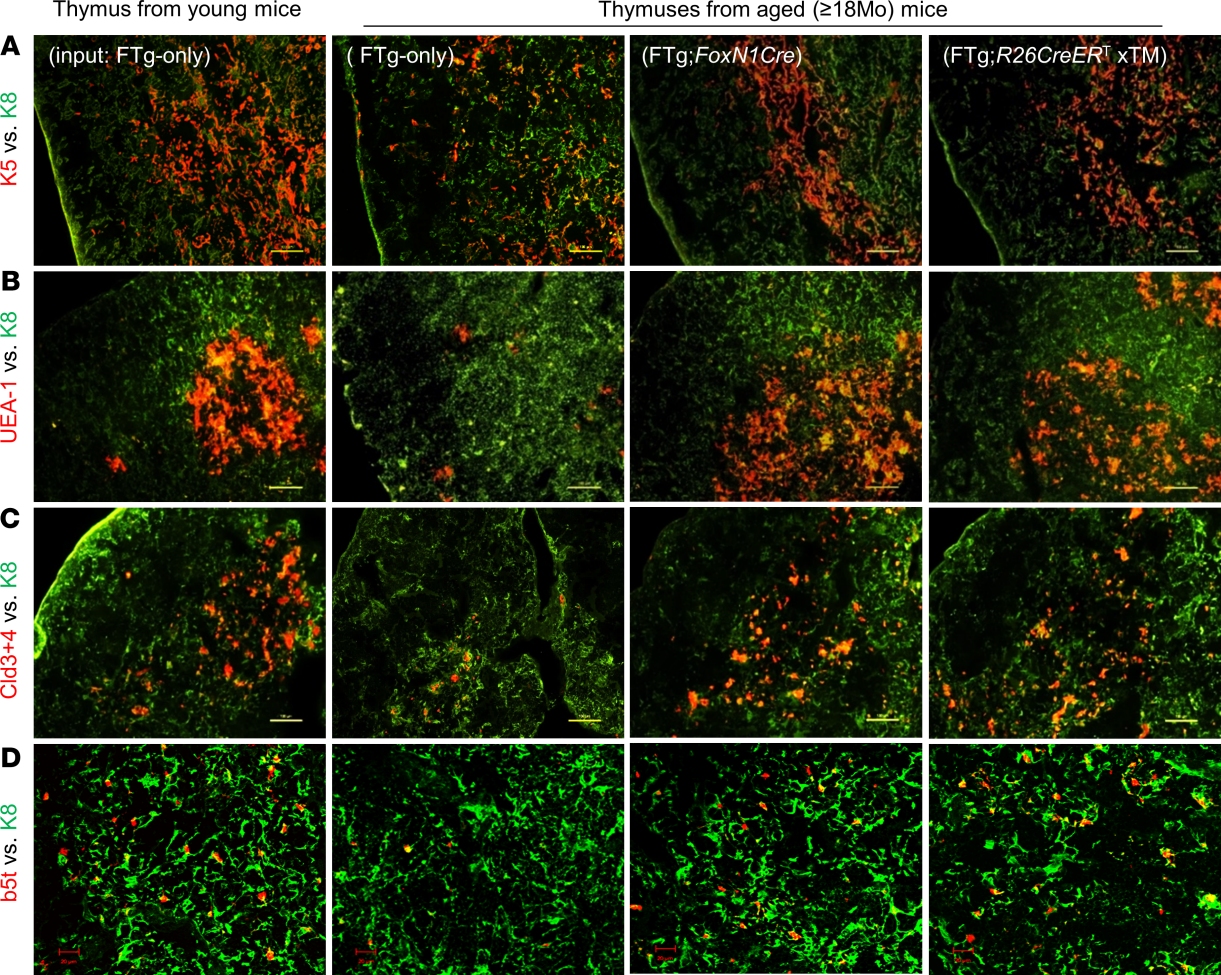
Transplantation of FREFs rejuvenated thymic architecture of aged mice. Same experimental setting as described in Figure 2. Cryosections of the thymic tissue (representative immunofluorescence images shown in **A**–**D**) were costained with various immunofluorescence antibodies for TEC developmental and architectural profiles. (**A**) K5 (red) vs. K8 (green), (**B**) UEA-1 (red) vs. K8 (green), (**C**) claudin-3 + claudin-4 (red) vs. K8 (green), and (**D**) β5t (red) vs. K8 (green). Data are representative of 3 biological replicates in each group with essentially identical results. Scale bars: 100 μm (**A**–**C**), 20 μm (**D**).

Increased thymic mass (Figure 2, A and B) generally reflects expansion in thymocytes (Figure 2C) and regrowth in TECs, because rejuvenation of TEC meshwork is essential for thymocyte regrowth. Therefore, we examined TEC-based thymic microstructure using TEC-associated markers (Figure 3). After costaining with keratin-5 (K5, red) (medullary region) and K8 (cortical region) (Figure 3, green), the aged, atrophied thymus showed disorganized and reduced K5^+^ regions (Figure 3A). After treatment with either FTg:*FoxN1Cre* or xTM FREFs (Figure 3A), the K5^+^ regions became organized, similar to the young thymus (Figure 3A). Increased UEA-1^+^ TECs showed the well-organized medulla exhibiting the same trends as the K5^+^ region (Figure 3B). Claudin-3 and -4 (Cld3+4) are immature medullary thymic epithelial cell (mTEC) markers (35, 36), and β5t is mainly expressed in immature cortical TECs (37). These were decreased in the naturally aged thymus, but were rescued in the naturally aged thymus treated with either of the 2 promoter-driven Cre-induced FREFs (Figure 3, C and D). These results imply that input of FREFs enhances native TEC regrowth to rejuvenate aged thymic architecture, thereby improving thymic microenvironment and rebooting thymopoiesis.

To confirm whether the observed TECs regrew from the native aged thymus when they received stimulation from an iTEC-rejuvenated microenvironment, or if these TECs grew directly from the newly transplanted FREFs, we examined the sources of these TECs in the rejuvenated, aged thymuses based on endogenous and exogenous FoxN1 expression. The TECs with positive staining for FoxN1 using rabbit anti-FoxN1 (antibody was provided by Manam itoi, Meiji University of Integrative Medicine, Kyoto, Japan) (38) represented only endogenous FoxN1, whereas the TECs with both antibody-specific FoxN1 staining and FTg-GFP (Supplemental Figure 1) expression (double positive) represented exogenous FoxN1 and, therefore, would be derived from the newly transplanted FREFs. We found that both native TECs and transplanted FREFs were expanding well within 10 days after the engraftment (Supplemental Figure 2B), particularly in mTECs (CD45^–^MHC-II^+^ population). Further, the transplanted FREFs exhibited reduced expansion, but the native TECs were still robustly expanding more than 20 days after the engraftment (Supplemental Figure 2B). The results suggest that although engrafted growth of FREFs is transient, they do exhibit growth and can also promote native TEC growth in the recipient thymus even after the growth of FREFs begins to wane. Thus, it seems that once native TECs receive necessary stimulation, they undergo a more prolonged expansion compared with the engrafted FREFs. However, both the engrafted FREFs and rejuvenated native TECs cooperate to restore the aged thymic microenvironment and promote thymocyte expansion.

### Engrafted FREFs expanded autoimmune regulator gene–expressing mTECs, increased negative selection signaling in CD4^SP^ thymocytes, and restored decreased thymocyte negative selection in the aged thymus.

**Figure 4:**
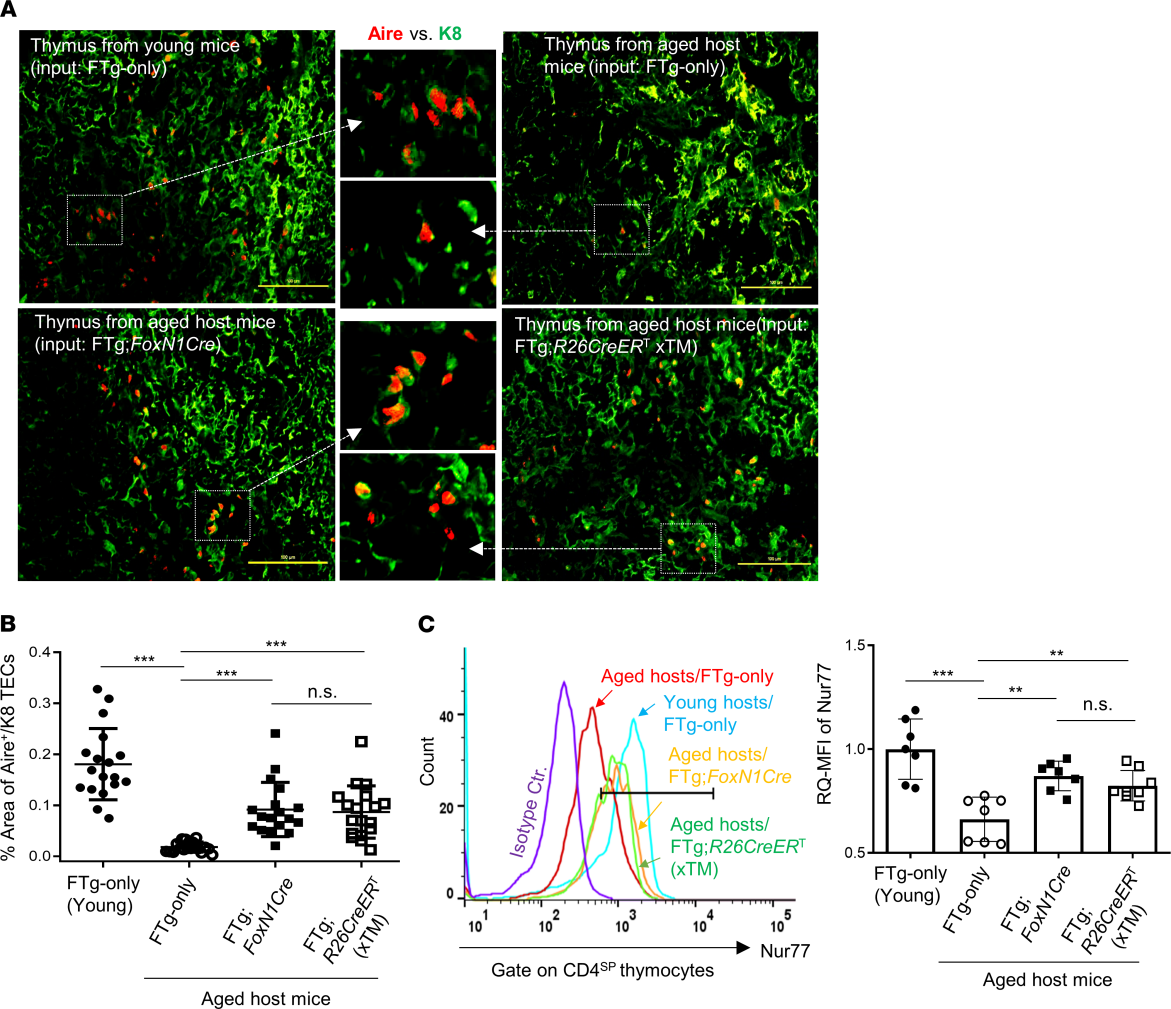
Transplantation of FREFs boosted *Aire* gene expression in the aged thymus and showed enhanced negative selection signaling strength via Nur77 in CD4^SP^ thymocytes of aged mice. Same experimental setting as described in Figure 2. (**A**) Representative immunofluorescence staining images of Aire^+^ TECs (red) in K8^+^ TEC counterstaining (green). Data are representative of 3 biological replicates in each group with essentially identical results. Scale bar: 100 μm. (**B**) Summarized result shows the percentage area of Aire^+^ TECs against K8^+^ counterstaining based on the slides in **A**. Each symbol represents 1 thymic tissue section; 5–7 of these thymic tissue sections per thymus at different physical locations (nonsequential slides) were counted using 3 thymuses per group and analyzed with ImageJ software (NIH; a total of 17–19 thymic tissue sections from 3 individual thymuses per group were observed). (**C**) Flow cytometric results show increased Nur77 signaling strength (relative quantitative [RQ] mean fluorescence intensity [MFI]) in CD4^SP^ thymocytes of young (control) or aged mice that were engrafted with MEFs or 2 types of FREFs. Left panel: histogram of Nur77 MFI in CD4^SP^ thymocytes; right panel: Nur77 RQ-MFI in CD4^SP^ populations of various groups (*n* = 7–8 mice per group). All results represent the mean ± SD. The statistical analysis was performed by 1-way Newman-Keuls multiple-comparisons test. **P < 0.01, ***P < 0.001. n.s., not significant.

The autoimmune regulator (Aire) gene is expressed by mTECs to mediate self-antigen expression and to promote central immune tolerance via thymocyte negative selection and Treg generation (39, 40). In the aged thymus, Aire-expressing mTECs are disrupted and/or decreased (7, 29). Because transplantation of FREFs enhanced biological characteristics of native TECs in the naturally aged thymus (Figure 3), we tested whether transplantation of the 2 FREF lines was able to expand the decreased Aire-expressing mTECs and found positive results (Figure 4A), with a *P* value of less than 0.001 (Figure 4B), in the aged thymus.

Self-autoreactive thymocytes undergo negative selection dependent on TCR signaling strength, whereas the intensity of Nur77 expression in thymocytes correlates with negative selection signaling strength. Therefore, we examined mean fluorescence intensity (MFI) of Nur77 in CD4 single-positive (CD4^SP^) thymocytes from various groups (Figure 4C), and found MFIs of Nur77 in CD4^SP^ thymocytes increased in the 2 FREF-grafted groups (Figure 4C). Although these increases did not reach the same levels as in young mice (Figure 4C), they significantly increased, compared with naturally aged controls (FTg-only group).

**Figure 5:**
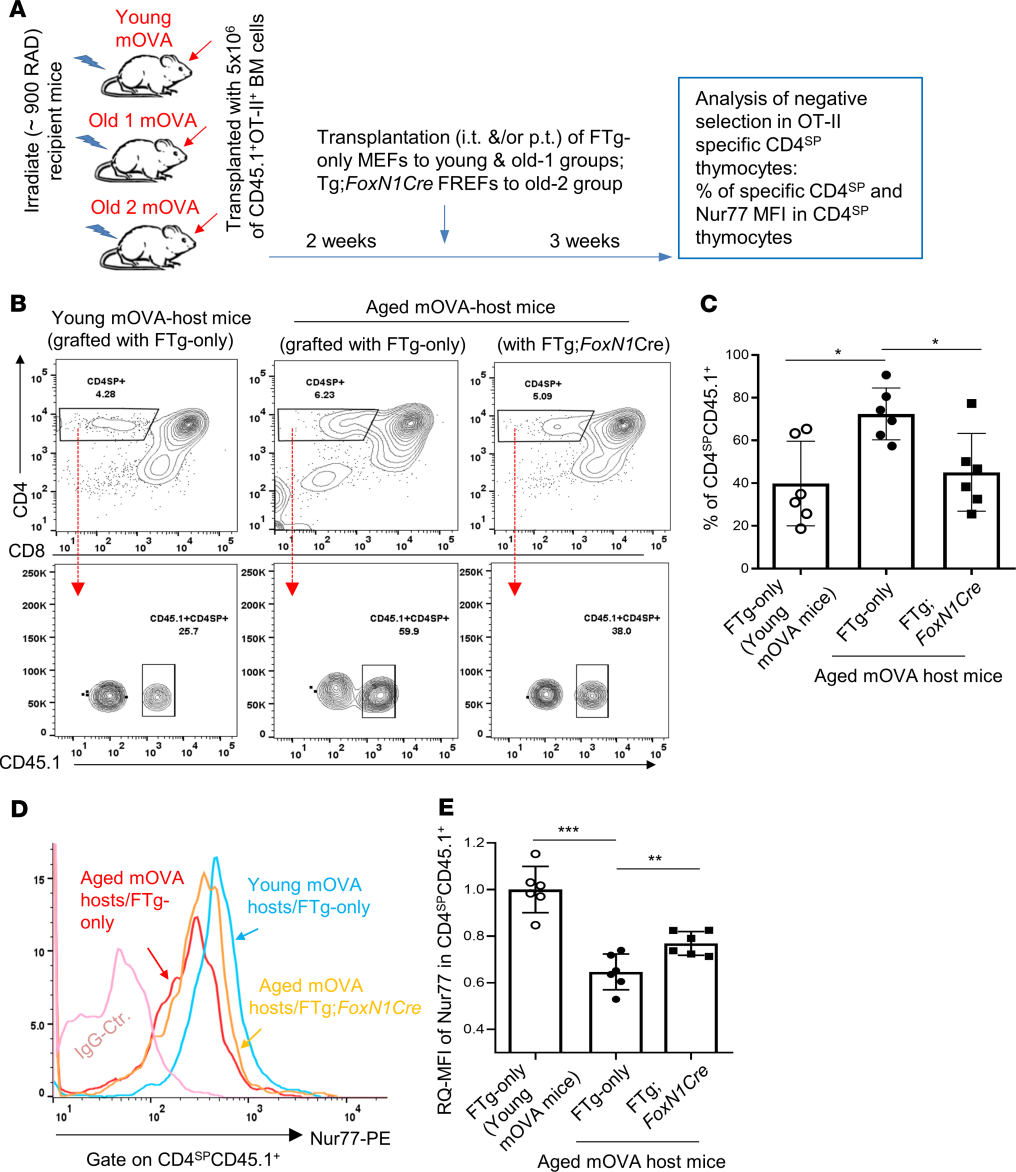
Transplantation of FREFs partially rescued decreased thymocyte negative selection in aged mice. (**A**) Reconstituted mOVA-Tg aged mice (mOVA-Tg young mice for control) with OT-II TCR-Tg bone marrow (BM) cells transplanted after approximately 900 Rad irradiation were intra-/perithymically transplanted with MEFs (FTg-only) or FREFs (FTg:*FoxN1Cre*). Negative selection of OT-II TCR-Tg–specific CD4^SP^ (CD4^+^CD8^–^) thymocytes in the host mOVA-Tg TEC microenvironment was analyzed with a flow cytometer. (**B**) Flow cytometric gating scheme of CD4 versus CD8 (top row) and engrafted donor BM (CD45.1^+^) produced OT-II–specific TCR-Tg CD4^SP^ thymocytes (bottom row). The histograms shown are representative of 3 independent experiments. (**C**) Summarized results of percent of OT-II–specific TCR-Tg CD4^SP^ thymocytes (*n* = 6 mice per group). (**D**) A representative histogram of Nur77 MFI in OT-II–specific TCR-Tg CD4^SP^ thymocytes. (**E**) RQ-MFI of Nur77 signaling strength in OT-II–specific TCR-Tg CD4^SP^ thymocytes, by setting RQ-MFI in young thymocytes as 1.0 (i.e., signaling with 100% intensity) (*n* = 6 mice per group). All results represent the mean ± SD compared with 1-way ANOVA Newman-Keuls multiple-comparisons tests. *P < 0.05, **P < 0.01, ***P < 0.001. n.s., not significant.

These results provided an indication that transplantation of FREFs potentially restores TEC function in negative selection as demonstrated by increased Aire^+^ mTECs and enhanced negative selection signaling strength in the CD4^SP^ thymocytes in the aged thymus. To obtain direct evidence that the decreased thymocyte negative selection in the aged thymus is actually restored, we designed an observable negative selection model, in which young and aged membrane-bound chicken ovalbumin driven by the rat insulin promoter–Tg (RIP mOVA-Tg) host mice were reconstituted with donor OT-II TCR-Tg mouse bone marrow (BM) cells. This is a well-designed, widely used thymocyte negative selection model, in which a neo–self-antigen, mOVA, presented on mTECs induces OT-II TCR-Tg CD4^SP^ thymocyte depletion (negatively selected), which is observable via flow cytometry assay (7, 41). The thymuses in the immune system–reconstituted young and aged mice were engrafted with FTg:*FoxN1*Cre FREFs or control FTg-only MEFs. Three weeks after the transplantation of these cells, the proportion of OT-II–specific CD4^SP^ thymocytes was determined (Figure 5A). Increased proportion of OT-II–specific CD4^SP^ thymocytes in the mOVA-Tg thymic microenvironment indicates defective negative selection, which was seen in the aged, atrophied thymus (Figure 5, B and C). However, this proportion was reduced after transplantation with FREFs (Figure 5, B and C) in the aged mOVA-Tg thymuses. Moreover, signaling of negative selection (Nur77) in the specific CD4^SP^ thymocytes was increased (Figure 5, D and E). These results suggest that engrafted FREFs were able to significantly restore mTEC-mediated function for self-reactive thymocyte negative selection in the aged, atrophied thymus.

### Engrafted FREFs counteracted inflammaging by reducing inflammatory cytokines and lymphocyte infiltration into a nonlymphoid organ in the periphery.

To confirm whether the restoration of negative selection in the FREF-engrafted aged thymus could counteract inflammaging-associated phenotypes in the aged periphery, we examined the levels of inflammatory cytokines and lymphocyte infiltration into nonlymphoid organs through adoptive transfer of spleen cells from rejuvenated mice. As previously reported, inflammaging is attributed not only to senescent somatic cells producing SASP and chronic innate immune cell activation, but also to self-autoreactive T cell–induced self-tissue damages. These self-reactive T cells are released from the aged, atrophied thymus owing to defective negative selection (5–8).

**Figure 6:**
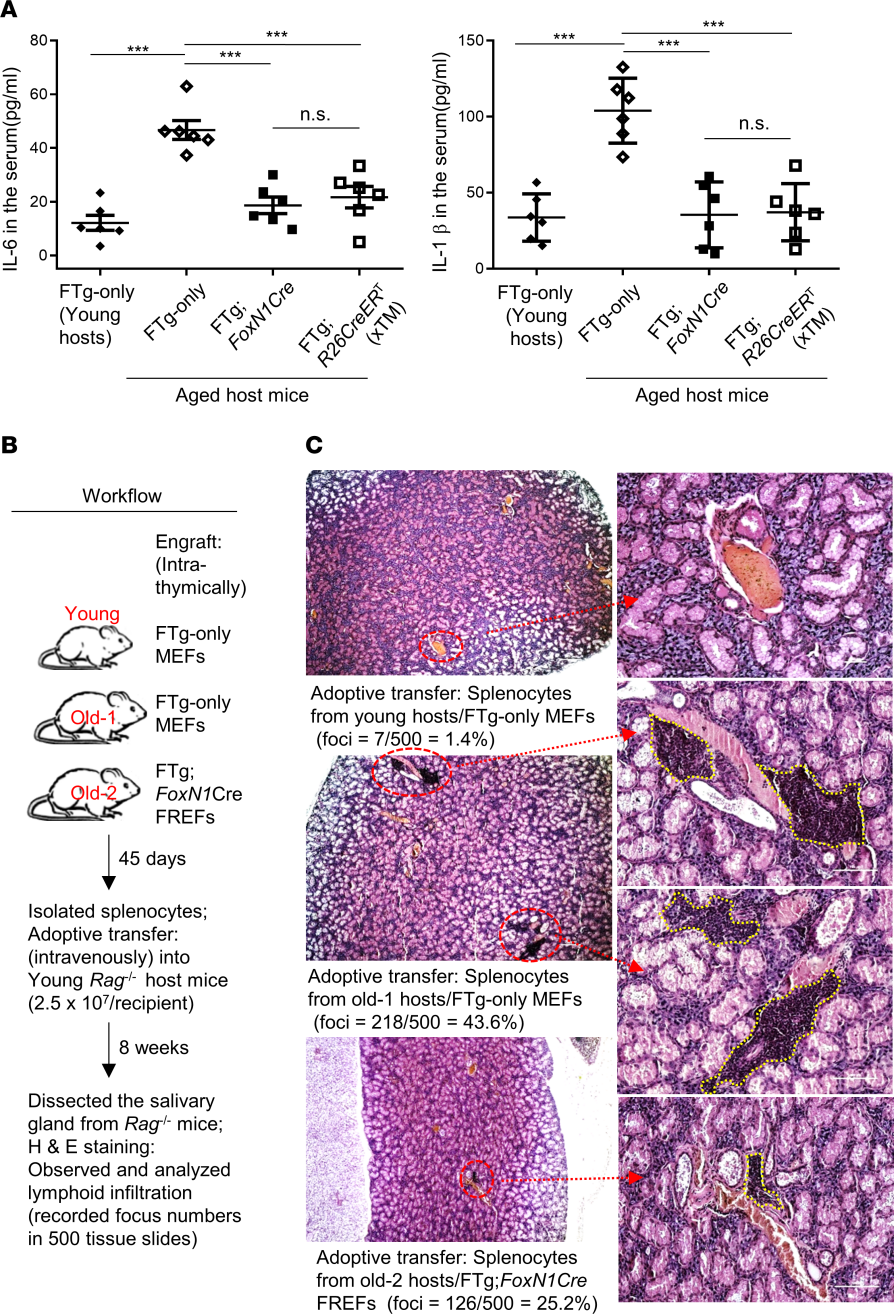
Transplantation of FREFs attenuated inflammaging-associated phenotypes by reducing inflammatory cytokines and lymphoid cell infiltration into a nonlymphoid organ in aged mice. (**A**) Serum was collected from mice with the same treatment as in Figure 2. Concentration of proinflammatory cytokines IL-6 (left panel) and IL-1β (right panel) in pg/mg of serum protein was measured through an ELISA method (*n* = 6 mice per group). All results represent the mean ± SD compared with 1-way ANOVA Newman-Keuls multiple-comparisons tests. ***P < 0.001. (**B**) Workflow of adoptive transfer, showing that splenocytes (2.5 × 10^7^ cells per recipient mouse) from rejuvenated and control young or aged WT mice were transferred via i.v. injection into young *Rag*^–/–^ recipient mice. Eight weeks after the transfer, the salivary glands were subjected to analysis of lymphocyte infiltration. (**C**) Representative H&E-stained images of the salivary glands from the adoptive transfer *Rag*^–/–^ recipient mice, showing foci of lymphoid cell infiltration (red circles in ×4 images and yellow circles in ×20 images). Data are representative of 500 tissue sections from 3 animals in each group (160–170 tissue sections per mouse), and numbers of infiltration foci in 500 tissue sections and the percentage of lymphoid cell infiltrated foci are shown.

We examined 2 inflammatory phenotypes. The first phenotype assessed consisted of 2 classic proinflammatory cytokines (IL-6 and IL-1β) in the serum of naturally aged mice 45 days after engraftment with FREFs or control MEFs. As previously reported, these cytokines increased in the serum of naturally aged mice (Figure 6A), but significantly decreased after engraftment with either type of FREF (Figure 6A).

The second phenotype assessed was lymphocyte infiltration into nonlymphoid tissue (the salivary gland). The approach was the same as previously described (7, 29), and the workflow is shown in Figure 6B. Splenocytes from thymus-rejuvenated mice or control mice were adoptively transferred into lymphocyte-free young *Rag*^-/-^ mice, and lymphocyte infiltration into the salivary gland (Figure 6C) was observed. Consistent with decreased inflammatory cytokines in the rejuvenated naturally aged mice (Figure 6A), we found that FREFs were able to reduce lymphocyte infiltration into the nonlymphoid tissue, salivary gland (Figure 6C, the bottom panels). The results indicate that engraftment of FREFs into the aged thymus rejuvenated thymic function, which in turn attenuated inflammaging-associated inflammatory phenotypes in aged mice.

### Engrafted FREFs indirectly reduced senescent T cells and enhanced T cell immune response in the periphery of aged mice.

**Figure 7:**
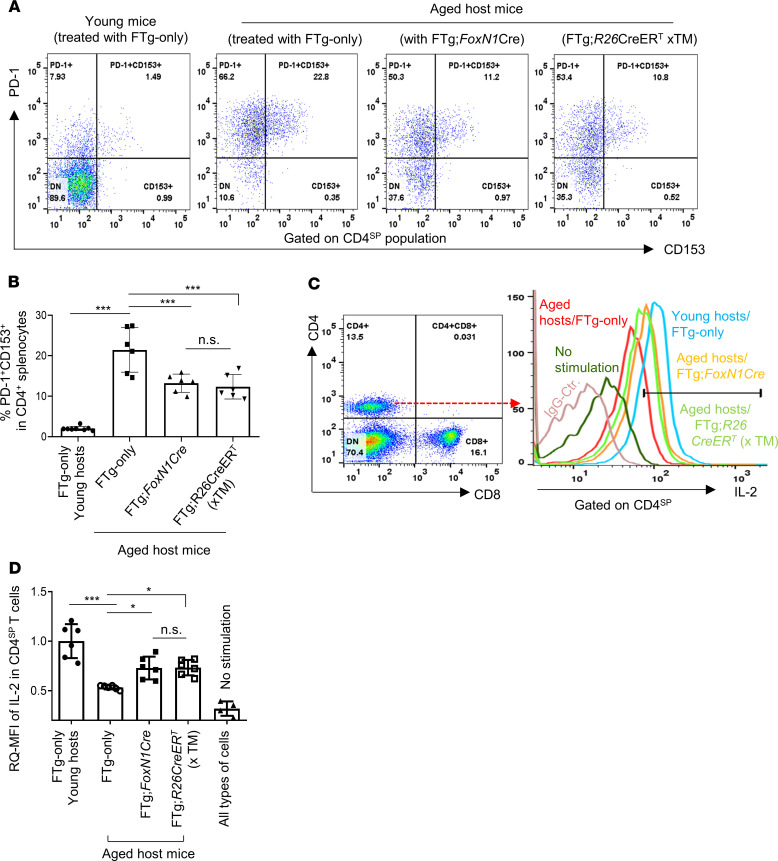
Transplantation of FREFs reduced senescent T cells and enhanced TCR response in the periphery of aged mice. Same experimental setting as described in Figure 2. Erythrocyte-depleted splenocytes were either directly stained for senescent T cells or isolated for culture (2 × 10^6^ per well) with costimulation of anti-CD3ε and anti-CD28 (2 μg/mL each) supplemented with GolgiSTOP (0.7 μL/mL, BD Biosciences) for 5 hours. (**A**) Flow cytometric gating scheme of senescent CD4^SP^ splenic T cells: PD-1^+^CD153^+^ (red boxes) in CD4^SP^ population. The histograms shown are representative of 3 independent experiments. (**B**) Summarized results of reduced senescent CD4^SP^ T cells after transplantation with either of 2 promoter-driven Cre-induced FREFs (right 2 bars), compared with aged mice treated with FTg-only MEFs (*n* = 6 mice per group). (**C**) Flow cytometric gating scheme of CD4^SP^ splenic T cell responses to the TCR costimulation of anti-CD3ε and anti-CD28: intracellular IL-2 (APC) levels in CD4^SP^ T cell population. (**D**) Summarized results of increased intracellular IL-2 levels in CD4^SP^ T cells of aged mice after transplantation with either type of FREFs (2 bars with filled or opened squares), compared with aged mice without FREF treatment (FTg-only). RQ-MFI, in which IL-2 MFI in young CD4^SP^ T cells (leftmost bar) was set as 1.0. (i.e., fully responsive to the costimulation) (*n* = 6 mice per group). All results represent mean ± SD compared with 1-way ANOVA Newman-Keuls multiple-comparisons tests. *P < 0.05, ***P < 0.001. n.s., not significant.

Inflammaging is also partially attributed to immunosenescence because senescent/exhausted peripheral T cells not only produce inflammatory factors, but are also unable to properly clear senescent somatic cells, which produce SASP (11, 42). We examined whether FREF-driven rejuvenation of aged thymic function could counteract inflammaging by reducing senescent T cells, considering that the rejuvenated thymus increases thymopoiesis (Figure 2). We found that 45 days after FREF engraftment, senescent CD4^SP^ T cells (CD4^+^PD-1^+^CD153^+^) (43, 44) were significantly reduced in the periphery of aged mice (Figure 7, A and B), compared with the aged mice that received FTg-only MEFs.

In addition, we also verified the peripheral CD4^SP^ T cell response to costimulation from CD3ε and CD28 antibodies. This response, represented by intracellular IL-2 MFI (Figure 7C), was decreased in peripheral CD4^SP^ T cells of aged mice (Figure 7D) (16), but was significantly restored in peripheral CD4^SP^ T cells from FREF-rejuvenated mice (Figure 7D), suggesting an increased proportion of newly generated T cells in the rejuvenated mice. Taken together, FREF-driven changes in the aged thymus could additionally confer positive rejuvenation effects on the peripheral T cell system.

## Discussion

T cell–mediated adaptive immunity during aging is intricately involved in both immunosenescence and inflammaging. One of the potential strategies for ameliorating these 2 extremes is rejuvenation of the aged, involuted thymus. Restoring thymic function of central tolerance establishment via repairing the defects in negative selection is critical for counteracting inflammaging. Although there are many strategies for rejuvenation of thymic involution, targeting defective TEC homeostasis via the *FOXN1*-TEC axis is one of the most relevant strategies studied to date.

We tested an application of cellular rejuvenation of age-related thymic involution by using FREFs generated from *R26*CreER^T^ and *FoxN1*Cre-induced exogenous *FoxN1* in FTg embryonic fibroblasts with intrathymic injection. We found that engrafted FREFs induced aged thymus regrowth, with increased thymopoiesis in aged male and female mice (Figure 2), in which native TECs were reorganized (Figure 3, A–D) and underwent expansion (Supplemental Figure 2B). These results suggest this restoration of aged thymus growth and thymic architecture occurs via both induction of endogenous TECs and exogenous pseudo-TECs driven from engrafted FREFs. Additionally, we observed reinforced thymocyte negative selection (Figure 4 and Figure 5). This resulted in reduced autoreactive T cell–mediated inflammaging-associated phenotypes and diminished peripheral senescent T cells in the aged periphery (Figure 6 and Figure 7).

The underlying mechanism of thymic rejuvenation potentially involves restoration of TEC regrowth in the aged thymus via both expansion of engrafted FREFs (increased GFP^+^FoxN1^+^, double-positive, TECs) and induced expansion of native TECs (increased GFP^–^FoxN1^+^, single-positive, TECs) (Supplemental Figure 2B). This improves the aged thymic microenvironment, promoting normalization of thymocyte homeostasis and development.

Because we observed that the engrafted FREFs restored the perturbed negative selection in the aged thymus, we assessed the effects on peripheral inflammaging-associated phenotypes. These outcomes culminated in attenuation of inflammaging phenotypes (Figure 6) and removal of senescent T cells (Figure 7, A and B). Although we did not determine the in-depth mechanisms by which CD4^+^PD-1^+^CD153^+^ senescent T cells were reduced in the periphery after FREF engraftment into the aged thymus, we believe that the rejuvenated thymus increases thymopoiesis (Figure 2), and that the newly generated naive T cells and the already existing peripheral T cells (both senescent and self-reactive) reach a new hemostatic balance. Additionally, we did not directly measure native T cells (T cells generated before FREF engraftment), but found that T cells from rejuvenated mice exhibited an increased response to TCR stimulation (Figure 7, C and D), which is a functional sign of healthy newly generated T cells.

Although the rejuvenation was partial, considering that it cannot be restored to the same levels as in young mice, it was significant when compared with age-matched counterparts treated with nonexogenous *FoxN1*-expressing MEFs. The effects of a onetime transplantation of these cells is also most likely transient, considering that the engrafted FREFs are not TEC stem cells and, therefore, do not demonstrate unlimited growth after engraftment in the aged, native thymus. Compared with the generation of an ectopic de novo thymus with induced *FOXN1*-overexpressing MEFs under the kidney capsule of adult mice (21) and intrathymic injection of newborn TECs into the middle-aged thymus (20), we believe that our strategy facilitates a more clinically translational rejuvenation therapy. Although an ectopic de novo thymus can generate naive T cells, this does not remedy the increased self-reactive T cells released by the native atrophied thymus remaining in the aged host. In addition, intrathymic injection of newborn TECs can rejuvenate the middle-aged thymus in mice (20), but the source of newborn TECs for human treatment is limited. Further, our rejuvenation effects were observed in aged mice (≥18 months old) rather than limited to middle-aged mice (20).

In comparison with exogenous *FoxN1* expression and rejuvenation effects from 2 promoter-driven *Cre*-Tg–mediated (*FoxN1Cre* and *R26CreER*^T^) FREFs, exogenous *FoxN1* expression was slightly higher in the former cell type (Figure 1C), and the effects were not that different between the 2 lines (Figure 2, Figure 3, and Figure 4). We believe that this is probably due to the length of time for which the exogenous *FoxN1*-Tg was activated. It had been turned on in vivo before their isolation, because endogenous *FoxN1*-driven Cre could have been already activated in the E13.5 MEF cells, whereas the exogenous *FoxN1*-Tg expression mediated by *R26*CreER^T^ was turned on after dissection and during the 48-hour culture with TM induction, i.e., 48 hours later than the former. However, the effector molecules (*Dll4* and *Ccl25*) most likely reach a homeostatic plateau. Once these 2 lines are injected into host mice, effector molecule expression in the *R26*CreER^T^ line could feasibly “catch up” to the levels expressed by the former line. In addition, *FoxN1Cre* mediated exogenous *FoxN1* expression only in skin epithelial cells of MEFs, whereas *R26CreER*^T^ mediated exogenous *FoxN1* expression in most tissues, including fibroblasts and epithelial cells derived from MEFs, considering that the *R26* promoter is ubiquitous. Therefore, it is not surprising that the effects from both cell lines are similar. These results suggest that this cellular therapeutic strategy is clinically translational, since fibroblasts derived directly from patients, who would be the treatment recipients, can be readily reprogrammed for genetic engineering of *FoxN1* expression. However, more research is required to determine whether human fibroblasts will respond with the same potential as the mouse fibroblasts used in this study.

In conclusion, our preliminary, proof-of-principle, cell-based rejuvenation strategy via the *FOXN1*-TEC axis with i.t./p.t. injection provides evidence for a promising thymic rejuvenation treatment with potential clinical significance. Once the application study is further formulated and investigated, i.t. transplantation of genetically reprogrammed *FoxN1-*expressing patient skin cells (fibroblasts) could facilitate attenuation of T cell immunosenescence and subclinical chronic inflammatory symptoms in elderly people. It should be noted, however, that the results from this research are within the confines of aged mice and that no research has been conducted on its application for human patients. Nevertheless, it is promising for further investigation for potential clinical treatments applications. In addition, some limitations of this study, such as the maximal length and distribution of grafted FREFs in the host thymus, the fate of exiting self-reactive T cells in the aged periphery after FREF engraftment, and other characteristics of translating aspects of FREFs to preclinical studies require further investigation.

## Methods

### Animal models.

C57BL/6 genetic background mouse models were used. WT young and aged mice were from our breeding colonies and National Institute on Aging aged rodent colonies. FTg mice were previously generated in our lab (31, 32) (Supplemental Figure 1) and crossbred with either *R26*-*CreER*^T^ mice (Jackson Laboratory 004847) or *FoxN1*-*Cre* mice (Jackson Laboratory 018448) for the generation of FTg:*R26CreER*^T^ (TM-inducible exogenous *FoxN1* overexpression in the *R26*-expressing tissues) and FTg:*FoxN1Cre* (exogenous *FoxN1* overexpression induced by endogenous *FoxN1* promoter-driven Cre-Tg, ref. 34) embryonic mice, respectively. Other genetically engineered mouse colonies included RIP-mOVA–Tg mice (Jackson Laboratory 005431), OT-II^+^ TCR-Tg (Tg TCR recognizing ovalbumin in the context of MHC-class II, I-A^b^) mice (Jackson Laboratory 004194), and *Rag*^–/–^ (*Rag1* gene KO) mice (45) (Jackson Laboratory 002216). Mouse ages are indicated in each figure legend or defined as young (1–2 months old) and naturally aged (≥18 months old).

### Preparation of MEFs for i.t. injection.

MEFs were prepared from E13.5 embryonic mice (the gestation day 0.5, “E0.5,” was determined by the presence of a vaginal plug in the first morning in the mother mouse). The head, limbs, and viscera of the embryonic mice were removed except for the trunk with skin, which was trypsinized with Trypsin-EDTA solution to generate single-cell suspensions. Cells were cultured in 10% FBS/DMEM medium, with 2 mM L-glutamine, 1 mM pyruvate, and 50 μM 2-ME. In cultured E13.5 embryonic FTg:*FoxN1Cre* MEFs (i.e., one type of iTEC donor cells), exogenous *FoxN1* is consistently expressed, owing to endogenous *FoxN1*-driven Cre having been turned on, which was found at part of the skin of E12.5 embryonic mice (34), and spontaneously activated at low levels, which was observed at E13.5 MEFs (21). For inducing exogenous *FoxN1* overexpression in FTg:*R26CreER*^T^ MEFs (i.e., another type of FREF donor cell), 1 μM 4-OHT was added in the culture for 48 hours. Exogenous *FoxN1* overexpression (based on GFP expression) in the 2 types of MEF lines was examined after 48 hours culture. These 2 cell lines were expanded with 2 passages. Third-passage cells, which were acquired after approximately 10 days of culturing, were used for injection. FTg-only (without any Cre Tg) MEFs (control donor cells) were used as a negative control. All embryonic mice for preparation of MEFs were genotyped. All cells were checked for GFP expression before engraftment.

### Intrathymic/perithymic injection of donor cells into recipient mice.

FTg-only MEFs (negative control) and 2 types of promoter-driven Cre-mediated FTg FREFs were injected at 1 × 10^6^ cells in 20 μL volume per recipient mouse (young or naturally aged) into the thymus and/or perithymus in 3 locations via a suprasternal notch surgery under anesthesia (46). Forty-five days after the injection, recipient mice tissues were analyzed for various phenotypes. More details about the surgical procedure are shown in Supplemental Methods.

### BM adoptive transfer for assessing negative selection.

Erythrocyte-depleted and mature T cell–depleted (via anti-CD3 MACS beads and columns, Miltenyi Biotec) BM cells from OT-II^+^ TCR-Tg mice, which carry a copy of the CD45.1 congenic marker, were i.v. injected into recipient young or aged mOVA-Tg mice at 5 × 10^6^ cells per recipient mouse, which had received irradiation at a dose of approximately 900 Rad. Two weeks after the BM cell transfer, FTg-only MEFs and FTg:*FoxN1Cre* FREFs were i.t. injected into the thymus and/or perithymus of the recipient mOVA-Tg mice. Three weeks after the engraftment, the thymuses of the recipient mOVA-Tg mice were dissected for analysis of negative selection (proportion of CD4^SP^ and MFI of Nur77 in CD4^SP^).

### Transplantation of splenocytes into Rag^–/–^ recipients for assessing lymphocyte infiltration.

Protocol per our previous study is as follows (7): briefly, erythrocyte-depleted splenocytes from FTg-only MEF- or FTg:*FoxN1Cre* FREF-engrafted young or aged WT mice were i.v. injected at 2.5 × 10^7^ cells per recipient mouse into the young recipient *Rag*^–/–^ mice. Eight weeks after the transplantation, the salivary glands from the young recipient *Rag*^-/-^ mice were analyzed for lymphocyte inflammatory infiltration with H&E staining in paraffin sections (5 μm thick).

### General analysis methods.

Detailed analysis methods (real-time RT-PCR, flow cytometer, immunofluorescence staining, and ELISA) and reagents are described in Supplemental Methods.

### Statistics.

Data were analyzed with Prism 8 software (GraphPad). Unpaired 2-tailed Student’s *t* test was used for comparisons between 2 groups, and ordinary 1-way ANOVA Newman-Keuls test was used for multiple comparisons. Data are shown as the mean ± SD. *P* values of less than 0.05 were considered significant.

### Study approval.

All animal experiments were performed in compliance with protocols approved by the Institutional Animal Care and Use Committee (IACUC) of the University of North Texas Health Science Center (IACUC-2016-0037), following guidelines of the NIH.

## Author contributions

JO designed the experiments and prepared the figures. JO and RT performed the experiments. WW, JO and DMS performed the hands-on animal work. RT proofread the manuscript. JO and DMS analyzed the data. JO, RT, and DMS wrote the manuscript. DMS conceived, designed, and supervised the entire project.

## Supplementary Material

Supplemental data

## Version 1 08/13/2020

In-Press Preview

## Version 2 09/17/2020

Electronic publication
