# Supplementary material for: Thymic rejuvenation via FOXN1-reprogrammed embryonic fibroblasts (FREFs) to counteract age-related inflammation
Source: JCI Insight. 2020 Sep 17;5(18):e140313. doi: 10.1172/jci.insight.140313 (PMC7526556; doi:10.1172/jci.insight.140313)
Supplement: Supplemental data [file jciinsight-5-140313-s132.pdf]

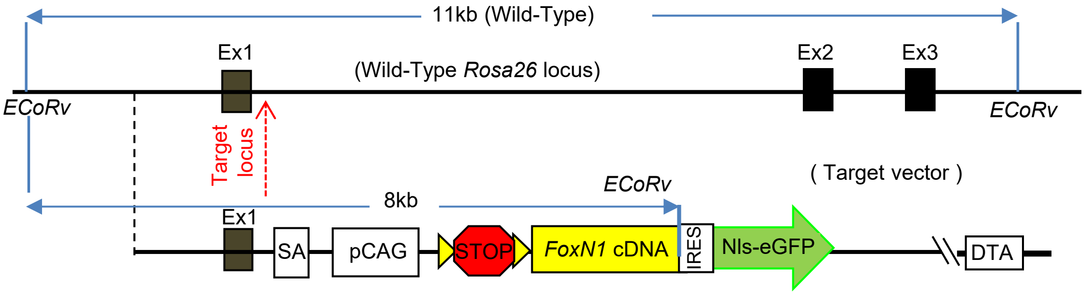

Figure S1. The generation of *Rosa26-STOP*^{flox}*-FoxN1*^{Tg} mice. Schematic diagram of *Rosa26-STOP*^{flox}*-FoxN1*^{Tg} gene targeting and elements of DNA insertion. Top line: Wild-type *Rosa26* locus; bottom line: insertional DNA in the target vector.

SA: splicing acceptor
pCAG: poly **C**MV enhancer element, chicken beta-**A**ctin promoter, and rabbit beta-**G**lobin splice acceptor site
STOP: DNA sequence cassette for transcriptional stop
Yellow triangles: two ***loxp***s
IRES: internal ribosome entry site
Nls-eGFP: nuclear localization signal (Nls) enhanced green fluorescent protein (eGFP).

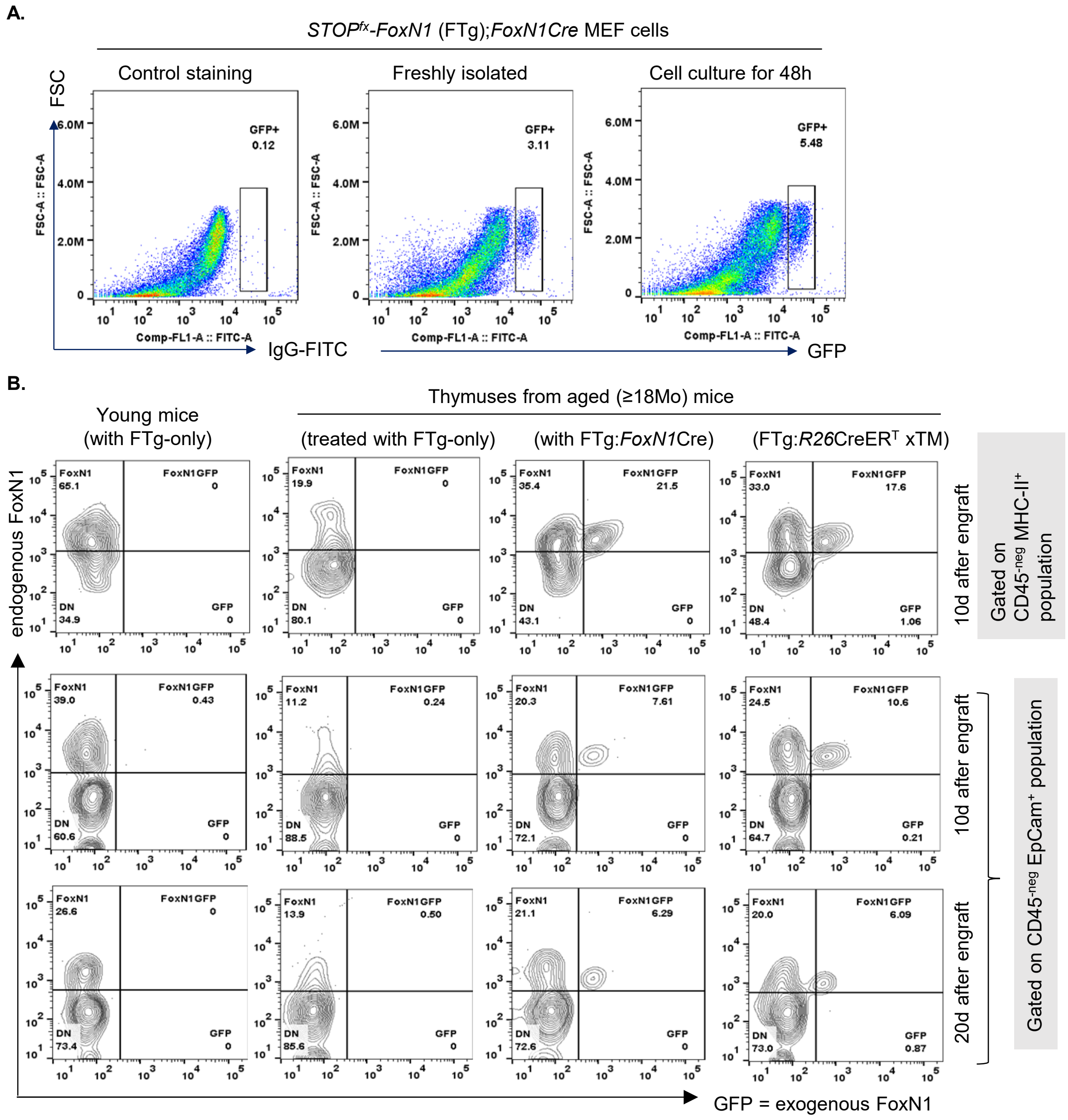

Figure S2. (**A**). *FoxN1Cre*-mediated activation of the *STOP^{fx}-FoxN1* (FTg). Freshly isolated *STOP^{fx}-FoxN1* (FTg);*FoxN1Cre* MEF cells (E13.5) were stained with IgG-FITC (control, left panel) and anti-GFP (middle panel), or cultured for 48 hours prior to staining with anti-GFP. The cells were analyzed by flow cytometer. (**B**). Endogenous TECs (from native thymus, FoxN1^{+}GFP^{-neg}) and exogenous TECs (from thymically injected FTg-FREFs, FoxN1^{+}GFP^{+}) in the thymus. Enzymatically dissociated thymic cells from young mice (left column) and aged mice (the other columns) were analyzed by flow cytometer, based on endogenous FoxN1 (by anti-FoxN1) and exogenous FoxN1 (by anti-GFP) expression. Endogenous TECs and exogenous TECs in the mTECs (gated on CD45^{-}MHC-II^{+}, top row) or pan-TECs (gated on CD45^{-}EpCam^{+}, middle and bottom rows) of various thymuses, 10 or 20 days after engraftment with MEF cells (FTg-only) or FTg-FREFs (FTg:FoxN1Cre or FTg:R26CreER^{T} x TM) were shown.

Thymic rejuvenation via *FOXN1*-reprogrammed embryonic fibroblast (FREF) to counteract age-related inflammation

Jiyoung Oh, Weikan Wang, Rachel Thomas, and Dong-Ming Su*

Department of Microbiology, Immunology, & Genetics, University of North Texas Health Science Center, Fort Worth, TX, 76107, USA.

Supportive Experimental Procedures

Real-time RT-PCR for gene expression

Total RNAs were isolated from FTg-only MEF cells or two types of FREF cells using TRIzol reagent (Invitrogen), then reversed transcribed (RT) into cDNA with the SuperScriptIII cDNA kit (Invitrogen/ThermoFisher Scientific). Real-time RT-PCR was performed with TaqMan reagents and primers of *FoxN1* (#Mm00433948), *Dll4* (Mm00444619), *Ccl25* (Mm00436443), and house-keeping gene, GAPDH, from Thermo Fisher scientific. The relative differences between samples were calculated using $\Delta\Delta C_T$ methods and GAPDH was used to normalize samples for comparison, as previously described (1).

Flow cytometry assays of single suspended cells

Single cells suspensions [homogenized thymocytes or splenocytes, or Collagenase-V/DNase-I dissociated TECs] were stained with various fluorochrome-conjugated antibodies (from BioLegend unless otherwise indicated) and analyzed with an LSR-II flow cytometer (BD Biosciences) and FlowJo software (1). For combinative cell surface and intracellular staining, cells were stained with multiple fluorochrome-conjugated antibodies on cell surface and blocked with

an Fc receptor antibody (CD16/CD32), followed by fixation/permeabilization solution (eBioscience, Cat. #88-8824-00), then intracellular staining, such as Nur77, IL-2, etc. (1).

Tissue sectioning with immunofluorescence

Dissected mouse tissues were prepared as 6μm-thick cryo-sections, and stained with various primary antibodies and fluorochrome-conjugated secondary antibodies as described previously (2-5). The results were visualized by a fluorescence microscope (Nikon Eclipse Ti-U) or confocal laser scanning microscope (LSM 510 Meta, Zeiss), and results were quantified by Image-J software.

ELISA assay for pro-inflammatory cytokines

Mouse serum was isolated from FREF cell-engrafted mice, and pro-inflammatory cytokines IL-6 and IL-1β were quantified by ELISA kit (BioLegend Cat. #431304 and #432605) (2). Each serum sample was diluted 1:2 with PBS and samples were prepared in duplicate. IL-6 and IL-1β standard concentration curves were observed within a range of 0-200 pg/ml. Absorbance was measured at 450nm with a BioTek ELx800 ELISA reader.

Image analyses

First, open the immunofluorescent staining overlaid image files (showing both red and green channels) in NIH Image-J software and split this merged double-channel immunofluorescent image into two single-channel images, then convert both images into binary images by clicking "Process". Click "Analyze" to measure positively stained cells in each layer in percentage (%).

Finally, the ratios of % positive areas (in our case, Aire positive staining) versus % counterstaining area (in our case, K8 positive staining) were calculated. One of the representative samples is shown in below.

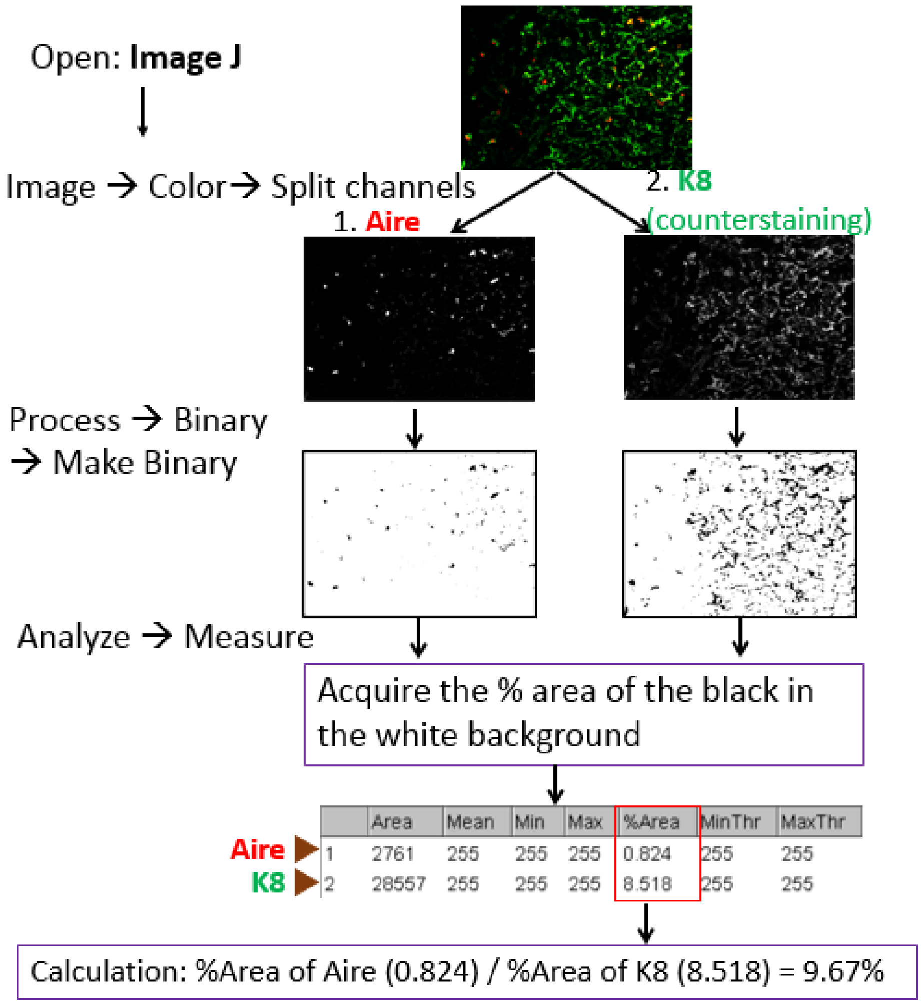

Surgical procedure for intra-/peri-thymic injection

Intra/peri-thymic injection is a routine technique in our Lab (3). Each anesthetized mouse was laid ventral side up and a sagittal ±0.5cm incision was cut with surgical scissors in the skin on the suprasternal fossa (below Figure-a). A pole was laid under the neck to allow for hyperextension of head. The muscle covering the suprasternal fossa was moved (below Figure-b, green arrow) with forceps to expose the suprasternal fossa (below Figure-b, red arrow). A Hamilton syringe was injected into the chest through the suprasternal fossa at almost 0° angle along the body toward the

tail. Two more injections were made by slightly shifting the needle angles toward the left and right of the body. The total volume injected per adult mouse was 25μl roughly equally distributed in the three differently angled injections. Lab members have practiced this operation many times with injection of trypan blue dye to perfect the accuracy of the injection in both normal thymus (below Figure-c left side) and atrophied thymus (below Figure-c right side).

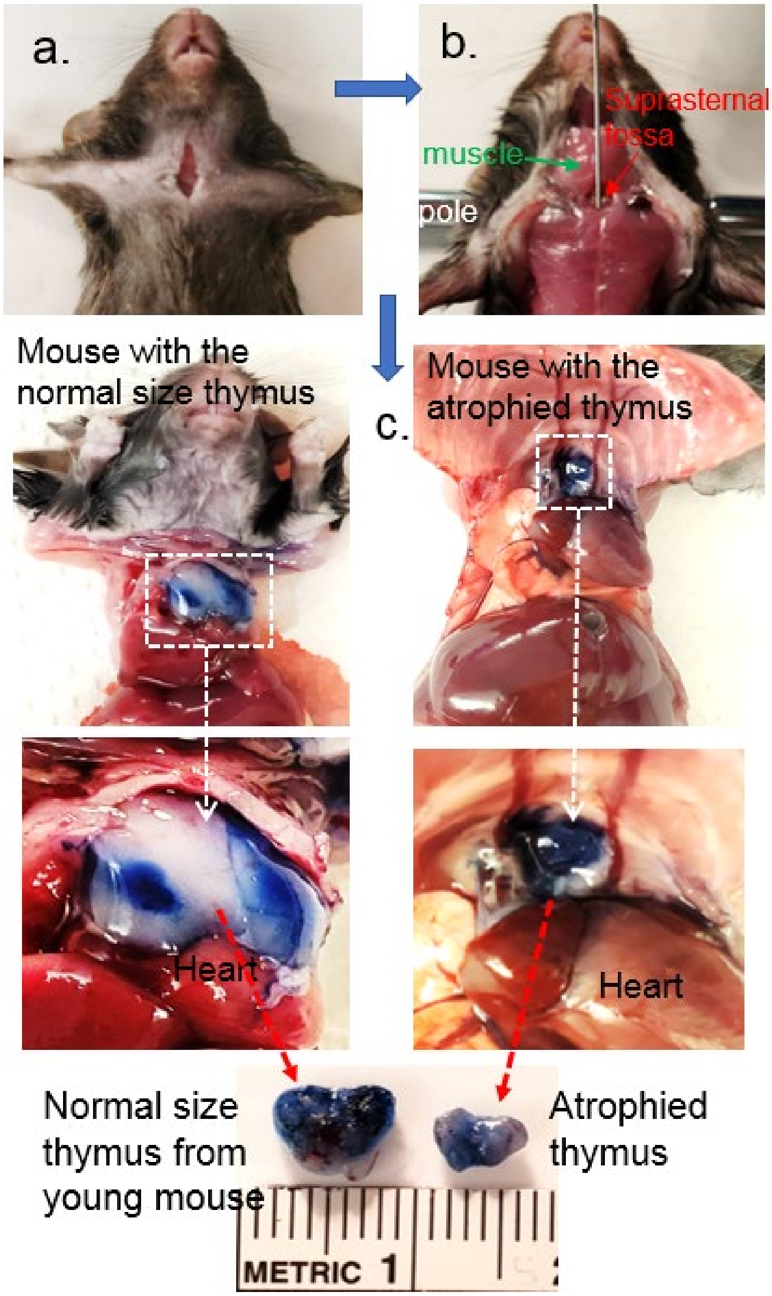

References

1. Oh J, Wang W, Thomas R, and Su DM. Capacity of tTreg generation is not impaired in the atrophied thymus. *PLoS Biol.* 2017;15(11):e2003352.
2. Sizova O, Kuriatnikov D, Liu Y, and Su DM. Atrophied Thymus, a Tumor Reservoir for Harboring Melanoma Cells. *Molecular cancer research : MCR.* 2018;16(11):1652-64.
3. Burnley P, Rahman M, Wang H, Zhang Z, Sun X, Zhuge Q, et al. Role of the p63-FoxN1 regulatory axis in thymic epithelial cell homeostasis during aging. *Cell Death Dis.* 2013;4:e932.
4. Ruan L, Zhang Z, Mu L, Burnley P, Wang L, Coder B, et al. Biological significance of FoxN1 gain-of-function mutations during T and B lymphopoiesis in juvenile mice. *Cell Death Dis.* 2014;5:e1457.
5. Wang W, Wang L, Ruan L, Oh J, Dong X, Zhuge Q, et al. Extracellular vesicles extracted from young donor serum attenuate inflammaging via partially rejuvenating aged T-cell immunotolerance. *FASEB journal : official publication of the Federation of American Societies for Experimental Biology.* 2018:fj201800059R.
